# Is cytotoxicity a determinant of the different in vitro and in vivo effects of bioactives?

**DOI:** 10.1186/s12906-017-1962-2

**Published:** 2017-09-07

**Authors:** Mattia Di Nunzio, Veronica Valli, Lidia Tomás-Cobos, Teresa Tomás-Chisbert, Lucía Murgui-Bosch, Francesca Danesi, Alessandra Bordoni

**Affiliations:** 1 Interdepartmental Centre for Industrial Agri-Food Research, University of Bologna, Piazza Goidanich 60, 47521 Cesena, Italy; 2 Department of Agri-Food Sciences and Technologies, University of Bologna, Piazza Goidanich 60, 47521 Cesena, Italy; 3 Department of Bioassays, Ainia Centro Tecnológico, Parque Tecnológico de Valencia, c. Benjamin Franklin 5-11, E46980 Paterna, Valencia Spain

**Keywords:** Cytotoxicity, Docosahexaenoic acid, Cyanidin-3-*O*-glucoside, Protocatechuic acid, Propionate, HepG2 cells

## Abstract

**Background:**

Foodstuffs of both plant and animal origin contain a wide range of bioactive compounds. Although human intervention studies are mandatory to assess the health effects of bioactives, the in vitro approach is often used to select the most promising molecules to be studied in vivo*.* To avoid misleading results, concentration and chemical form, exposure time, and potential cytotoxicity of the tested bioactives should be carefully set prior to any other experiments.

**Methods:**

In this study the possible cytotoxicity of different bioactives (docosahexaenoic acid, propionate, cyanidin-3-*O*-glucoside, protocatechuic acid), was investigated in HepG2 cells using different methods. Bioactives were supplemented to cells at different concentrations within the physiological range in human blood, alone or in combination, considering two different exposure times.

**Results:**

Reported data clearly evidence that in vitro cytotoxicity is tightly related to the exposure time, and it varies among bioactives, which could exert a cytotoxic effect even at a concentration within the in vivo physiological blood concentration range. Furthermore, co-supplementation of different bioactives can increase the cytotoxic effect.

**Conclusions:**

Our results underline the importance of in vitro cytotoxicity screening that should be considered mandatory before performing studies aimed to evaluate the effect of bioactives on other cellular parameters. Although this study is far from the demonstration of a toxic effect of the tested bioactives when administered to humans, it represents a starting point for future research aimed at verifying the existence of a potential hazard due to the wide use of high doses of multiple bioactives.

## Background

Food bioactives are both nutrients (i.e., peptides and polyunsaturated fatty acids) and extra-nutritional constituents that typically occur in small quantities in foods and have positive effects on human health [1]. They vary widely in chemical structure and function and are grouped accordingly [2].

Although food bioactives have shown potential health benefits, currently there are no specific recommended intakes (i.e., Dietary Reference Intakes, DRIs) for the most of them. DRIs are based on requirements for the specific nutrient to maintain normal physiologic or biochemical function, and to prevent signs of deficiency and adverse clinical effects; for some nutrients, they have been expanded to include criteria for reducing the risk of chronic degenerative diseases [3]. The evaluation of the beneficial properties, the effective dose and the safety of bioactives are essential for establishing the corresponding DRIs, and this represents a big challenge to many scientists.

In vitro studies are often performed to establish the effectiveness and mechanism of action of bioactives, but the scientific uncertainties of the extrapolation of in vitro data to humans justifies the European Food Safety Authority’s (EFSA) mandatory requirement for human intervention studies before health claim approval. Notwithstanding, in preliminary studies the use of cell culture is convenient and advantageous, one obvious benefit being the reduction of animal use and the involvement of human beings [4]. Despite the wide use of cell cultures for the determination of bioactive effectiveness and mechanism of action, three important scientific gaps are seldom considered:

- in vivo, foods are digested and some bioactives are extensively metabolized so that the effective molecules could be very different from parent compounds [5, 6]. Many studies reporting the protective effect of specific bioactives have been performed supplementing cells with putative active compounds in the form they are present in the food, not in the human body;
- the bioactive concentration used for cell supplementation should be similar to that reachable in vivo in the circulatory system, which is nmol/L to low mmol/L. In many studies cells have been exposed to super-physiological bioactive concentrations;
- cell cultures represent a close system and their direct and continuous exposure to bioactives could alter the cell response, inducing cytotoxicity due to the lack of the continuous detoxification and clearance of compounds occurring in the whole body. In in vitro studies bioactive concentrations that are physiological in vivo could induce cytotoxicity, and alter the cell response to bioactive compounds. Cytotoxicity plays an important role in a number of pathological processes, including carcinogenesis and inflammation. It may also modulate the activity of other agents, including free radicals, irritants, and genotoxins [4]. In this light, the time of cell exposure to bioactives should be carefully considered as well.

Hence, in in vitro studies it is fundamental to set the bioactive concentration and chemical form and the time of exposure prior to any other experiments concerning effectiveness [7].

The aim of this study was to assess in vitro the time and concentration related-cytotoxicity of four different food bioactives having important implications for human health. Since the liver is the organ mainly involved in xenobiotic metabolism [8], the human hepatoma HepG2 cell line was used as model system. HepG2 cells are widely used in biochemical and nutritional studies, and are considered one of the experimental models that more closely resembles the primary cultured hepatocytes [9].

Bioactives to be tested were selected based on their differences in term of food source, chemical form, rate of absorption and metabolism, and in vivo physiological concentration in human blood.

(i). C22:6 n-3 docosahexaenoic acid (DHA) is a long-chain omega-3 fatty acid. DHA is absorbed by intestinal cells and it is delivered to peripheral cells in its parent form. Although the bioavailability of DHA is subject to considerable variability, DHA plasma concentration being up to 600 μM 6 h after a meal rich in n-3 polyunsaturated fatty acids (PUFAs) [10], 50–200 μM is considered as reference range of DHA plasma levels [11–14]. Accordingly, cultured cells were firstly supplemented with DHA at 200 μM concentration.
(ii). propionate (PRO) is a short-chain fatty acid (SCFA) derived from the colonic microbiota fermentation of fibers [15]. In vivo, the majority of the SCFAs by fiber fermentation are absorbed by the caecum and the colon, where they are used as an energy source by colonocytes [16]. In contrast to butyrate, the majority of PRO produced in the colon is absorbed, passes the colonocytes and the viscera, and drains into the portal vein [17, 18]. Around 90% of absorbed PRO is metabolized by the liver. PRO concentration in human peripheral blood ranges from 1 to 10 μM [19–22], while portal concentration lies between 17 and 194 μM [23]. Since roughly 75% of the blood entering the liver is venous blood from the portal vein, PRO was first supplemented to cultured hepatic cells at 100 μM concentration.
(iii). cyanidin-3-*O*-glucoside (C3G) is one of the most representative phenolic compound in anthocyanin-rich foods, and its concentration in plasma is low [24]. The C3G physiological range in plasma is 1–140 nM [25–29]. Accordingly, cytotoxicity was first assessed supplementing cells with 140 nM C3G concentration;
(iv). protocatechuic acid (PCA) is the main metabolite of the most anthocyanins [28, 29]. PCA concentration in human plasma is in the range 0.1–10 μM [25, 28, 30–32], so first experiments were performed using the 10 μM PCA concentration.

When testing a compound for cytotoxicity many biological endpoints can be considered, and simple tests are needed to identify at a first glance the general aspects of cellular toxicity. In the past, a number of methods have been developed to study different parameters associated with cell death and proliferation in cell culture [33]. In the herein reported study, four different assays i.e., cell count, MTT, Alamar Blue (AB), and trypan blue (TB) exclusion were selected and compared. All these methods measure a biochemical event that occurs in living cells, and stops after cell death, using indicator dyes that undergo a change in physical properties [34].

Since toxicity could be induced without any sign of cellular damage [35], in some experiments cell clonogenic capacity and *BAX* (BCL2 associated X protein) and *BCL2* (B-cell CLL/lymphoma 2) gene expression ratio were also evaluated. The clonogenic assays or colony formation assay consists of an in vitro cell survivor method based on the ability of single cultured cells to grow into colonies consisting of at least 50 cells. It therefore detects all cells that have retained the capacity for producing a large number of progeny after possibly damaging treatments [36]. *BAX* and *BCL2* are cell survival related genes encoding for proteins of the Bcl2 family, thought to be principal participants in a cellular decision-making process regarding whether a cell in a specific context will live or die [37]. In particular Bcl-2 and Bax proteins have been associated respectively to anti-apoptotic and pro-apoptotic functions, and the ratio between these two antagonists determines the susceptibility of a cell to apoptosis [38].

The possible cytotoxic effects of the selected compounds were first evaluated after 24 h exposure to the highest concentration within the above reported physiological range in the human plasma. According to the obtained results, other concentrations were then tested. Cytotoxicity was also assessed after 48 h exposure to the bioactive concentrations not evidencing any sign of toxicity after the shorter exposure time (24 h). Furthermore, bioactives were tested in combination.

Results herein reported clearly show that the toxicity limit depends on the type and concentration of the test agent, the time of exposure, and the assay used, and underline the importance of cytotoxicity screening before performing in vitro studies.

## Methods

### Chemicals

Dulbecco’s modified Eagle’s medium (DMEM) and Dulbecco’s phosphate-buffered saline (DPBS) were purchased from Lonza (Basel, Switzerland). C3G was from Polyphenols Laboratories AS (Sandnes, Norway). All other chemicals, reagents, and solvents were purchased from Sigma-Aldrich Co. (St. Louis, MO, USA), unless otherwise stated. All aqueous solutions were prepared using ultrapure water (Milli-Q; Millipore, Bedford, CT, USA).

### Cell culture and bioactive compounds supplementation

HepG2 human hepatoma cells were maintained at 37 °C, 95% air, 5% CO_2_ in DMEM supplemented with 10% (*v*/v) fetal bovine serum (FBS), 100 U/mL penicillin and 100 mg/mL streptomycin (P/S). Once a week, cells were split 1:20 into a new 75 cm^2^ flask, and medium was refreshed [39]. Cells were seeded in 12-well plates at 0.6 × 10^6^ cells/mL concentration. After 24 h, at 75–80% confluence, cells were divided randomly into six groups and incubated for 24 or 48 h with the different bioactive compounds.

DHA was dissolved in 100% ethanol, and complexed to bovine serum albumin (BSA); fatty acid–BSA complexes were prepared fresh each time at a final BSA concentration of 0.5% in serum-free DMEM. PCA was dissolved in dimethyl sulfoxide (DMSO) acidified with HCl (at pH 2), while PRO was dissolved in water. Control cells received corresponding amounts of BSA, ethanol and DMSO. The final concentration of ethanol and DMSO was kept below 0.1% in serum-free DMEM. After 24 or 48 h incubation with the bioactive compounds cells were washed twice with warm DPBS and cell viability and number evaluated in each well.

### Viability and cell number

Cell viability was assessed using the methylthiazolyldiphenyl-tetrazolium bromide (MTT) assay as reported by Valli et al. (2012) [40] with slight modifications. Cells were washed twice with warm DPBS, then MTT dissolved in RPMI-1640 medium (final concentration 0.5 mg/mL) was added to cells. After 1 h at 37 °C, medium was completely removed, 0.5 mL n-propanol were added to dissolve the formazan product, and the absorbance measured against a propanol blank at 560 nm using a microplate reader (Tecan Infinite M200; Tecan, Männedorf, Switzerland). Cell viability was expressed as percentage of the viability of control cells, assigned as 100%.

In experiments performed in cells supplemented with the single bioactives, cell viability was also assessed using the Alamar Blue (AB) assay [41, 42]. Cells were incubated 18–24 h at 37 °C in the dark with AlamarBlue® Cell Viability Reagent (Life Technologies Ltd.; Paisley, UK) in an amount equal to 10% of the culture volume. Fluorescence was measured at λ_excitation_ = 540 nm and λ_emission_ = 590 nm using the fluorescence plate reader Fluostar Optima (BMG Labtechnologies; Offenburg, Germany).

The number of total and viable cells was also determined by staining cell populations with trypan blue (TB) [43]. Cells were washed twice with warm DPBS, incubated with trypsin–EDTA for 2 min to remove adherent cells, and suspended in DMEM supplemented with 10% (*v*/v) FBS. Ten μL of suspended cells were mixed with an equal part of TB (final concentration 0.2%) and cells viability and number were determined using a TC20 Automated Cell Counter (Bio-Rad Laboratories; Hercules, CA, USA). Cell viability was expressed as percentage of the total cells.

### Colony count

Clonogenic assay was performed according to Buch et al. (2012) [44] with some modifications. After 24 h supplementation, cells were washed twice with warm DPBS and incubated with 200 μL of trypsin–EDTA for 2 min. After the addition of 800 μL complete growth medium (DMEM plus FBS plus P/S), cells were collected, diluted 1: 10 and counted using a TC20 Automated Cell Counter (Bio-Rad). Cells were then seeded in 6-well plates at 1500 cells/mL, and allowed to grow and form colonies for 11 days. Medium was refreshed every 72 h. On day 11 medium was removed, and cells were washed twice with warm DPBS. Cells were then fixed and stained adding a mixture of 0.5% crystal violet in 50:50 methanol:water (1 mL/well) for 30 min under shaking. Dishes were rinsed with water and allowed to dry at room temperature. Colonies were visualized with ChemiDoc™ MP (Bio-Rad Laboratories; Hercules, CA, USA) equipped with a white light conversion screen; images were acquired with Image Lab™ Software. The counting of the colonies was performed using ImageJ software [45]. Clonogenic capacity was expressed as survival fraction as previously reported by Franken et al. (2006) [36].

### *BAX/BCL2* gene expression ratio

Total RNA was automatically isolated by Maxwell® 16 system (Promega; Mannheim, Germany), and cDNA was obtained by the High Capacity cDNA Reverse Transcription Kit (Applied Biosystems; Foster City, CA, USA). Real-time polymerase chain reaction (qPCR) was carried out on a 7300 Real-Time PCR System, using the TaqMan® chemistry with commercial primers and probes (*BAX*, Hs01016552_g1; *BCL2*, H Hs00608023_m1) (Applied Biosystems; Foster City, CA, USA). Gene expression was normalized to the reference gene β-actin (4326315e). Data were analyzed with 7300 System SDS Software (Applied Biosystems; Foster City, CA, USA), and are presented as mean fold change of expression compared to control cells, normalized to one.

### Statistical analysis

Resulting data on and fatty acid composition, lipid accumulation and cholesterol concentration are given as mean ± standard deviation (SD). The statistical differences were determined by the one-way analysis of variance (ANOVA) followed by Dunnett’s test for comparison with control cells, considering *p* < 0.05 as significant.

## Results

### Single bioactive supplementation

**Fig. 1:**
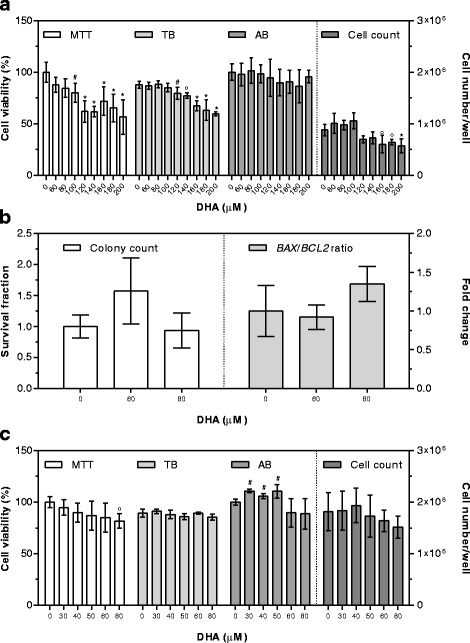
Cytotoxic effects of DHA after 24 (**a**, **b**) and 48 (**c**) hours of supplementation. Cell viability by MTT and AB is expressed as percentage of control cells (assigned as 100%). TB is expressed as percentage of total cells. Cell count is expressed as number of cells per well. Colony count by clonogenic assay is expressed as survival fraction. *BAX/BCL2* gene expression ratio is expressed as the mean fold change of relative expression compared to control cells, normalized to one. Data are means ± SD of six samples obtained from two independent experiments. Statistical analysis was by the one-way ANOVA (panel **a**: MTT and cell count *p* < 0.001, TB *p* < 0.05, AB n.s.; panel **b**: colony count n.s., *BAX/BCL2* ratio n.s.; panel **c**: MTT, TB, and AB *p* < 0.05, cell count n.s.) using Dunnett’s as post-test to compare supplemented cells to control ones (# *p* < 0.05; ° *p* < 0.01; * *p* < 0.001)

After 24 h exposure, the first DHA concentration used to assess cytotoxicity (200 μM) negatively influenced both cell viability (by MTT and TB assays) and cell count (Fig. 1a). Therefore, seven lower DHA concentrations (180, 160, 140, 120, 100, 80 and 60 μM) were tested. No significant differences in cell number/well were detected up to 140 μM DHA concentrations, while the percentage of viable cells significantly decreased at 120 μM DHA concentration by the TB method, and at 100 μM DHA concentration by MTT assay (Fig. 1a). No differences were detected using the AB assay.

To further substantiate these findings at the apoptotic level, cell clonogenic ability and *BAX*/*BCL2* gene expression ratio were evaluated using the two highest bioactive concentrations showing no detrimental effects in the previous experiments. No modifications in colony formation or *BAX*/*BCL2* ratio were detected (Fig. 1b).

DHA cytotoxicity was then assessed after 48 h exposure, using the two highest concentrations showing no toxic effects in any test after 24 h exposure (80 and 60 μM). In addition, three lower concentrations (50, 40 and 30 μM) were tested. No significant modifications in cell number/well and cell viability by the TB method were detected at any concentration, while cell viability evaluated by the MTT method significantly decreased at 80 μM DHA concentration. The AB assay showed an increased viability in cells supplemented with 30–60 μM DHA (Fig. 1c).

**Fig. 2:**
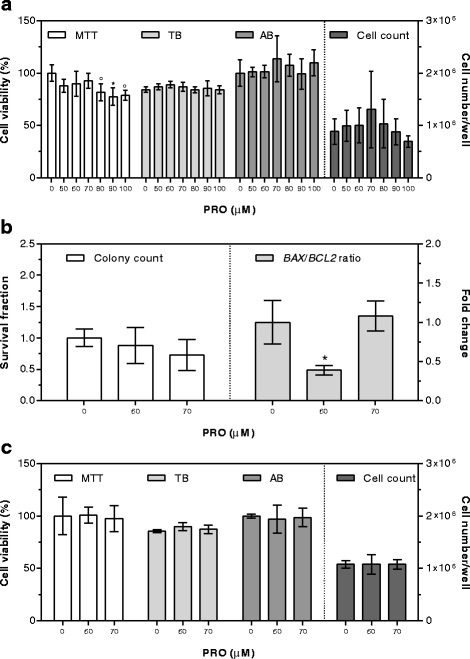
Cytotoxic effects of PRO after 24 (**a**, **b**) and 48 (**c**) hours of supplementation. Cell viability by MTT and AB is expressed as percentage of control cells (assigned as 100%). TB is expressed as percentage of total cells. Cell count is expressed as number of cells per well. Colony count by clonogenic assay is expressed as survival fraction. *BAX/BCL2* gene expression ratio is expressed as the mean fold change of relative expression compared to control cells, normalized to one. Data are means ± SD of six samples obtained from two independent experiments. Statistical analysis was by the one-way ANOVA (panel **a**: MTT *p* < 0.001, TB, AB, and cell count n.s.; panel **b**: colony count n.s., *BAX/BCL2* ratio *p* < 0.001; panel **c**: MTT, TB, AB, and cell count n.s.) using Dunnett’s as post-test to compare supplemented cells to control ones (° *p* < 0.01; * *p* < 0.001)

PRO cytotoxicity was first assessed using the 100 μM concentration for 24 h. Cell viability by TB and AB assays and cell count were not affected by the supplementation, while the MTT method evidenced a significant decrease in cell viability (Fig. 2a). So, five lower PRO concentrations (90, 80, 70, 60, and 50 μM) were tested. Again, the MTT method was the only one evidencing a cytotoxic effect starting at 80 μM PRO concentration (Fig. 2a).

To further exclude cytotoxicity of PRO concentrations lower than 80 μM, cell clonogenic ability and *BAX*/*BCL2* gene expression ratio were evaluated at PRO 70 and 60 μM concentration. Compared to control cells, supplemented ones showed neither decrease in clonogenic ability nor increase in susceptibility to apoptosis, which appeared significantly lower after supplementation with 60 μM PRO (Fig. 2b).

The 60 and 70 μM concentrations were then used to assess PRO cytotoxicity after 48 h exposure, and no significant modifications were detected at any concentration using the different tests (Fig. 2c).

**Fig. 3:**
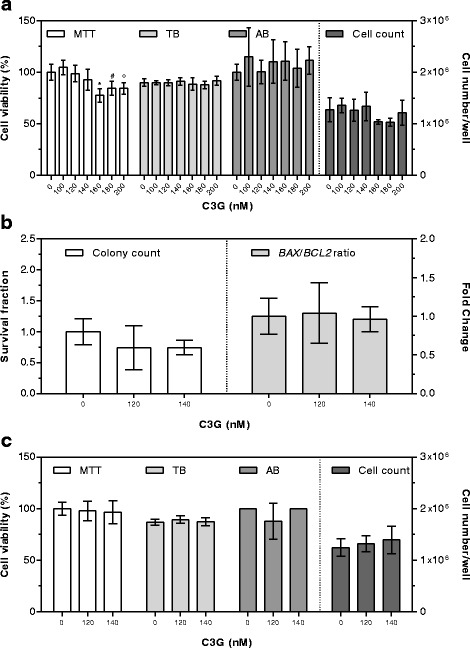
Cytotoxic effects of C3G after 24 (**a**, **b**) and 48 (**c**) hours of supplementation. Cell viability by MTT and AB is expressed as percentage of control cells (assigned as 100%). TB is expressed as percentage of total cells. Cell count is expressed as number of cells per well. Colony count by clonogenic assay is expressed as survival fraction. *BAX/BCL2* gene expression ratio is expressed as the mean fold change of relative expression compared to control cells, normalized to one. Data are means ± SD of six samples obtained from two independent experiments. Statistical analysis was by the one-way ANOVA (panel **a**: MTT *p* < 0.001, TB, AB, and cell count n.s.; panel **b**: colony count n.s., *BAX/BCL2* ratio n.s.; panel **c**: MTT, TB, AB, and cell count n.s.) using Dunnett’s as post-test to compare supplemented cells to control ones (# *p* < 0.05; ° *p* < 0.01; * *p* < 0.001)

No significant modifications in cell number/well and cell viability were detected after 24 h exposure using the C3G 140 nM concentration (Fig. 3a). Accordingly, three higher (160, 180, and 200 nM) and two lower C3G concentrations (120 and 100 nM) were then tested. The MTT assay evidenced a decrease in cell viability at concentrations higher than 140 nM, while no differences compared to controls were detected using other assays (Fig. 3a).

The two highest concentrations showing no cytotoxic effects (120 and 140 nM) were then used to evaluate the clonogenic ability and *BAX*/*BCL2* gene expression ratio, that appeared comparable to controls (Fig. 3b).

The 120 and 140 nM C3G concentration were then tested after 48 h exposure, and no significant modifications in cell number/well and percentage of viable cells were detected (Fig. 3c).

**Fig. 4:**
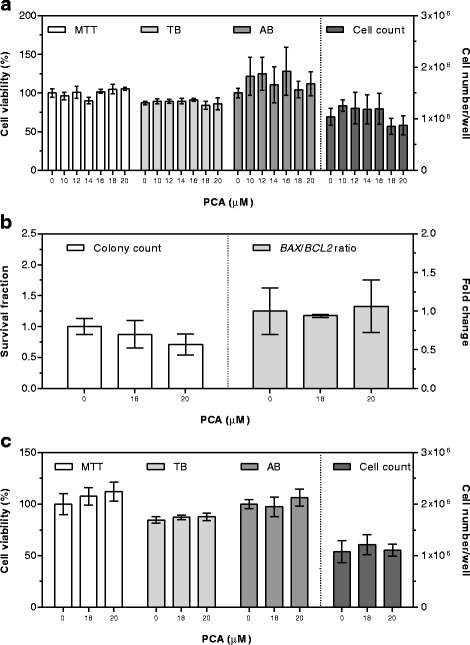
Cytotoxic effects of PCA after 24 (**a**, **b**) and 48 (**c**) hours of supplementation. Cell viability by MTT and AB is expressed as percentage of control cells (assigned as 100%). TB is expressed as percentage of total cells. Cell count is expressed as number of cells per well. Colony count by clonogenic assay is expressed as survival fraction. *BAX/BCL2* gene expression ratio is expressed as the mean fold change of relative expression compared to control cells, normalized to one. Data are means ± SD of six samples obtained from two independent experiments. Statistical analysis was by the one-way ANOVA (panel **a**: MTT, TB, AB, and cell count n.s.; panel **b**: colony count n.s., *BAX/BCL2* ratio n.s.; panel **c**: MTT, TB, AB, and cell count n.s.) using Dunnett’s as post-test to compare supplemented cells to control ones (n.s.)

PCA cytotoxicity was first assessed using the 10 μM concentration for 24 h. Since no sign of toxicity was detected at this concentration (Fig. 4a), five higher concentrations (12, 14, 16, 18, and 20 μM) were tested, and neither modifications in cell number/well nor in cell viability were evidenced (Fig. 4a). As well, the two highest PCA concentrations showed no effect on clonogenic ability and *BAX*/*BCL2* gene expression ratio (Fig. 4b).

PCA cytotoxicity was then assessed after 48 h exposure using the two highest concentrations showing no toxicity after the shorter exposure time (18 and 20 μM). Again, no significant modifications were detected at any concentration used (Fig. 4c).

### Combined bioactive supplementations

To point out possible synergism or antagonism, in the second part of the study bioactives were supplemented to cells in combination. Different combinations were considered: i. C3G plus PCA; ii. DHA plus other bioactives.

In experiments evaluating C3G plus PCA cytotoxicity, the six highest non-toxic C3G concentrations were used, maintaining a C3G: PCA ratio = 1:100. Accordingly, combinations tested for cytotoxicity ranged from 140 nM C3G: 14 μM PCA to 90 nM C3G: 9 μM PCA.

**Fig. 5:**
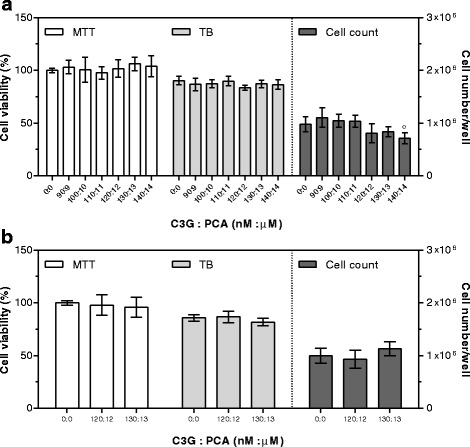
Cytotoxic effects of C3G plus PCA after 24 (**a**) and 48 (**b**) hours of supplementation. Cell viability by MTT is expressed as percentage of control cells (assigned as 100%). TB is expressed as percentage of total cells. Cell count is expressed as number of cells per well. Data are means ± SD of six samples obtained from two independent experiments. Statistical analysis was by the one-way ANOVA (panel **a**: cell count *p* < 0.01, MTT and TB, n.s.; panel **b**: MTT, TB, and cell count n.s.) using Dunnett’s as post-test to compare supplemented cells to control ones (° *p* < 0.01)

After 24 h, cell number/well significantly decreased using the 140 nM C3G: 14 μM PCA supplementation, while no significant modifications in cell viability were detected at any concentration (Fig. 5a). C3G plus PCA cytotoxicity was also assessed after 48 h exposure using the two highest concentrations showing no toxicity after the shorter exposure time (130 nM C3G: 13 μM PCA and 120 nM C3G: 12 μM PCA), and no significant modifications were detected (Fig. 5b).

To evaluate the effects of DHA combined with other bioactives, three concentrations were chosen: i. the highest concentration showing no toxicity after 48 h supplementation (DHA = 60 μM; PRO = 70 μM; PCA = 20 μM; C3G plus PCA = 130 nM + 13 μM, respectively); ii. the lowest concentration within the physiological range in human plasma (DHA = 50 μM; PRO = 1 μM; PCA = 0.1 μM; C3G plus PCA = 1 nM + 0.1 μM, respectively) [12, 19, 32]; iii. The average of the previous two (DHA = 55 μM; PRO = 35 μM; PCA = 10 μM; C3G plus PCA = 65 nM + 6.5 μM, respectively).

**Fig. 6:**
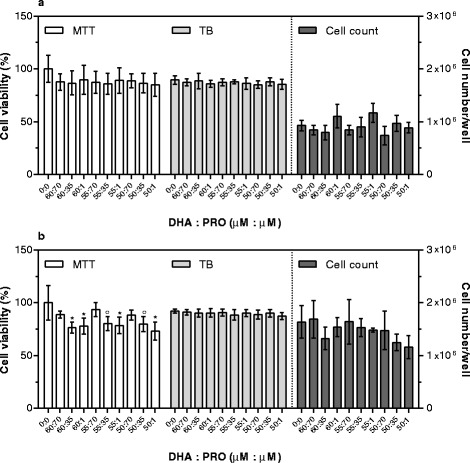
Cytotoxic effects of DHA plus PRO after 24 (**a**) and 48 (**b**) hours of supplementation. Cell viability by MTT is expressed as percentage of control cells (assigned as 100%). TB is expressed as percentage of total cells. Cell count is expressed as number of cells per well. Data are means ± SD of six samples obtained from two independent experiments. Statistical analysis was by the one-way ANOVA (panel **a**: MTT, TB, and cell count n.s.; panel **b**: MTT *p* < 0.001, TB, and cell count n.s.) using Dunnett’s as post-test to compare supplemented cells to control ones (° *p* < 0.01; * *p* < 0.001)

**Fig. 7:**
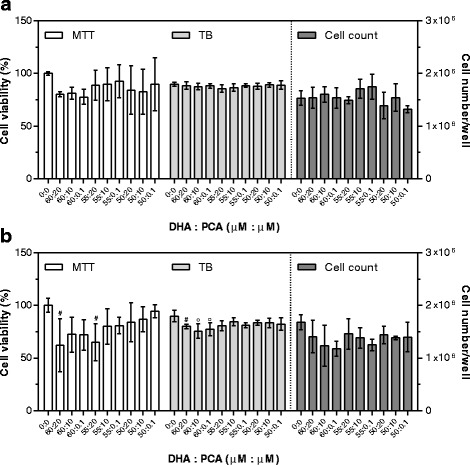
Cytotoxic effects of DHA plus PCA after 24 (**a**) and 48 (**b**) hours of supplementation. Cell viability by MTT is expressed as percentage of control cells (assigned as 100%). TB is expressed as percentage of total cells. Cell count is expressed as number of cells per well. Data are means ± SD of six samples obtained from two independent experiments. Statistical analysis was by the one-way ANOVA (panel **a**: cell count *p* < 0.01, MTT *p* < 0.001, TB n.s; panel **b**: MTT *p* < 0.05, TB *p* < 0.05, cell count n.s.) using Dunnett’s as post-test to compare supplemented cells to control ones (# *p* < 0.05; ° *p* < 0.01)

**Fig. 8:**
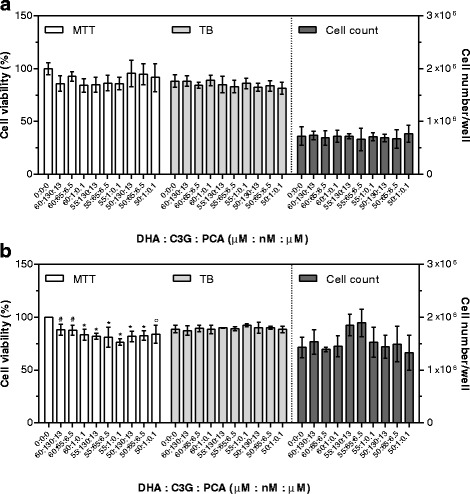
Cytotoxic effects of DHA plus C3G plus PCA after 24 (**a**) and 48 (**b**) hours of supplementation. Cell viability by MTT is expressed as percentage of control cells (assigned as 100%). TB is expressed as percentage of total cells. Cell count is expressed as number of cells per well. Data are means ± SD of six samples obtained from two independent experiments. Statistical analysis was by the one-way ANOVA (panel **a**: MTT, TB, and cell count n.s.; panel **b**: MTT *p* < 0.001, TB, and cell count n.s.) using Dunnett’s as post-test to compare supplemented cells to control ones (# *p* < 0.05; ° *p* < 0.01; * *p* < 0.001)

After 24 h supplementation, no significant differences in cell number/well and cell viability were detected in cells supplemented with DHA in combination with other bioactives (Figs. 6a, 7a, and 8a). As well, after 48 h supplementation no modifications in cell number were detected in any combination and concentration used (Figs. 6b, 7b, and 8b). Using the TB assay, no significant differences were detected in cells supplemented with DHA plus PRO, and DHA plus C3G plus PCA. On the contrary, in cells supplemented with DHA plus PCA viability decreased when the highest DHA concentration was used, independent of PCA concentration. The MTT assay revealed a decreased viability in all DHA plus PRO supplemented cells except the ones receiving the highest PRO concentration, in cells supplemented with 60 or 55 μM DHA plus 20 μM PCA, and in all cells supplemented with DHA plus C3G plus PCA.

## Discussion

The main aim of the present study was to evidence the possible cytotoxic effects of different bioactives, supplemented alone or in combination to cultured hepatic cells. Different cytotoxicity assays were used to detect early cytotoxic events, so increasing the reliability of the data and avoiding over- or underestimations due to the different sensitivity of the methods themselves. Bioactives were first supplemented at concentrations corresponding to the highest physiological concentration found in human plasma, then other concentrations were tested according to the obtained results. Once the highest concentrations with no toxic effects after 24 h exposure were selected, they were tested also after a longer exposure time (48 h).

In our experimental conditions, PRO supplementation at concentration up to 70 μM, which is achievable in human portal blood [20] but is 7 times above the highest concentration found in the systemic circulation, did not show any cytotoxicity either after 24 or 48 h exposure. These results are in line with previous findings showing that PRO exerts cytotoxic effects only at millimolar concentrations [46], probably related to autophagy associated with a decreased mechanistic target of rapamycin (mTOR) activity and enhanced AMP-activated protein kinase (AMPK) activity [47].

As well, no cytotoxic effects after both 24 and 48 h exposure were detected in cells supplemented with C3G alone and PCA alone at concentrations equal (C3G 140 nM) or even higher (PCA 20 μM) than the corresponding highest concentration found in human blood. Our results are in agreement with a previous study indicating that C3G has pro-apoptotic and anti-proliferative effects at concentrations ≥10 μM [48]. Although PCA is the main metabolite that is formed after anthocyanin-rich food consumption [28], small C3G amounts, generally <1%, of intake, are present in plasma in the native form [28, 49, 50]. Therefore, in the present study the possible cytotoxicity of combined supplementation with C3G and PCA was also assessed, maintaining a C3G: PCA ratio = 1:100. It is worth noting that the co-supplementation of C3G and PCA had an additive effect on the cytotoxic effects of the single compounds. It is worth noting that these effects could be not only due to C3G and PCA themselves, but also to newly-formed metabolites within the cell model. Detailed investigation of the compounds derived from anthocyanins in hepatic cells is still lacking in literature, as recently observed by Aragonès et al. (2017) [51]. Regardless consideration about a direct or not direct cytotoxicity due to C3G and PCA supplementation, the additive effect of co-supplementation deserve attention.

Comparing the physiological range in human blood to the results obtained in the first part of the present study (Table 1), DHA appeared the bioactive having the highest cytotoxic potential in vitro even when supplemented within the in vivo physiological range. Fatty acids are known to influence cell proliferation both in vivo and in vitro [52], and in vitro studies evidenced that n-3 and n-6 PUFAs at micromolar concentrations are toxic to several cell lines [53]. The mechanisms responsible for the cytotoxic effect of DHA have not been completely defined, but published evidence supports the hypothesis that this fatty acid may induce cytotoxicity with different mechanisms including reactive oxygen species (ROS) formation [54], alteration of plasma membrane fluidity [55], interference with cell signaling [56] and accumulation of neutral lipid droplets in the cytosol [57]. Although lipoperoxidation is considered one of the most probable cause of DHA cytotoxic effect in various cell lines [58], in a previous work we evidenced that 60 μM DHA supplementation to HepG2 cells did not cause lipid peroxidation. On the contrary, it induced the intracellular antioxidant defenses [59]. DHA cytotoxicity could be related to the selective incorporation of the fatty acid in the different lipid classes. DHA supplemented to primary cultures of rat cardiomyocytes is esterified in phospholipids (PLs) and triacylglycerols [60]. The consequent higher unsaturation of membrane PLs may alter lipid rafts and their environment [61, 62], thereby modulating the in vitro potential cytotoxicity of DHA.

**Table 1:** Summary of the highest bioactive concentrations found in human plasma compared to the highest non-cytotoxic ones detected in the herein reported in vitro study

Bioactive	Highest concentration found in vivo	Highest non-cytotoxic concentration observed in vitro (24 h exposure)	Highest non-cytotoxic concentration observed in vitro (48 h exposure)
DHA	200 μM [11–14]	80 μM	60 μM
PRO	10 μM (systemic circulation) [19–22]; ≈200 μM (portal circulation) [23]	70 μM	70 μM
C3G	140 nM [25–29]	140 nM	140 nM
PCA	10 μM [25, 28, 30–32]	20 μM	20 μM

To verify possible additive or antagonist effects of co-supplemented bioactives, DHA was also supplemented to cells in combination with PRO, PCA, and C3G plus PCA. Combined supplementation of DHA and C3G was not tested, since in vivo C3G is metabolized to PCA, thus resulting in the appearance of PCA or PCA plus C3G in human plasma, and not C3G alone [28, 63].

After 24 h exposure, co-supplementation of DHA and other bioactives did not cause detrimental consequences, indicating the absence of additive effects.

On the contrary, when co-supplementation lasted for 48 h an additive effect was clearly evidenced. According to Vauzour et al. (2015) [64], the additive effect of C3G and PCA cannot be ascribed to an increased DHA intracellular concentration due to enhanced DHA synthesis from precursors. As well, in a recent study we observed neither an increased DHA concentration in hepatic cells supplemented with PRO, nor an additional increase of DHA concentration in cell supplemented with DHA plus PRO compared to DHA alone [65].

Notably, the increase in DHA cytotoxicity was not evident when PRO was co-supplemented at the highest concentration. This antagonist effect cannot be ascribed to competition in uptake between the two fatty acids, since in mammalian cells long chain fatty acid uptake is strictly regulated by CD36, plasma membrane-associated fatty acid-binding protein (FABP), and family of fatty acid transport proteins (FATPs) [66], while short-chain fatty acids mainly diffuse across plasma membrane or enter into cells with a monocarboxylate transporter-dependent mechanism [67].

## Conclusions

In summary, our results clearly evidence that some bioactives exert cytotoxic effects in vitro even at a concentration within the in vivo physiological blood concentration range. Our understanding of the molecular mechanism behind the observed effects is still limited, and to further explore them is out of the scope of this study. Our aim was to demonstrate that bioactives cytotoxic concentration not always overlaps in vitro and in vivo*,* and to underline the importance of in vitro cytotoxicity screening. In order to avoid misleading results, cytotoxicity screening should be considered mandatory before performing studies aimed to evaluate the effect of bioactives on other cellular parameters.

In addition, our results point out that bioactive toxicity is tightly related to the exposure time, and the combined supplementation of different bioactives, considered a promising approach to optimize the dietary strategies against many diseases, could generate synergism/antagonism having both positive [68] and detrimental effects.

From a methodological point of view, results herein reported indicate AB as the less sensitive, and MTT as the most sensitive assay among the ones used to detect cytotoxicity, as previously suggested by Fotakis and Timbrell (2006) [69]. The high sensitivity of the MTT assay was also verified using two additional assays aimed to verify an apoptotic effect that could be induced without any sign of cellular damage. Bioactive concentrations negative to MTT assay did not increase cell susceptibility to apoptosis, confirming that this method is fast, inexpensive and very sensitive.

The authors are aware that the herein reported data are far from the demonstration of a toxic effect of the tested bioactives when administered to humans. The aim of the study was to highlight the not complete overlapping between in vitro and in vivo cytotoxic concentrations. Bioactive consumption with the diet appears to be safe. Notwithstanding, unresolved issues related to toxicity still remain when bioactives are supplemented to humans in high, concentrated doses and in combination, and must be seriously addressed. Although our results do not solve this important concern, they could represent a starting point for future research aimed to verify the existence of a potential hazard due to the wide use of high doses of multiple bioactive compounds.

## Abbreviations

AB: Alamar blue
AMPK: AMP-activated protein kinase
ANOVA: Analysis of variance
BAX: BCL2 associated X protein
BCL2: B-cell CLL/lymphoma 2
BSA: Bovine serum albumin
C3G: Cyanidin-3-*O*-glucoside
CD36: Cluster of differentiation 36
cDNA: Complementary DNA
DHA: Docosahexaenoic acid
DMEM: Dulbecco’s modified Eagle’s medium
DMSO: Dimethyl sulfoxide
DPBS: Dulbecco’s phosphate-buffered saline
DRI: Dietary reference intake
EDTA: Ethylenediaminetetraacetic acid
EFSA: European Food Safety Authority
FABP(pm): Plasma membrane-associated fatty acid-binding protein
FATP: Family of fatty acid transport protein
FBS: Fetal bovine serum
mTOR: Mechanistic target of rapamycin
MTT: Methylthiazolyldiphenyl-tetrazolium bromide
n.s.: Not significant
P/S: Penicillin and streptomycin
PCA: Protocatechuic acid
PL: Phospholipid
PUFA: Polyunsaturated fatty acid
qPCR: Real-time polymerase chain reaction
RNA: Ribonucleic acid
ROS: Reactive oxygen species
SCFA: Short-chain fatty acid
SD: Standard deviation
TB: Trypan blue

## References

[1] Kris-Etherton PM, Hecker KD, Bonanome A, Coval SM, Binkoski AE, Hilpert KF, Griel AE, Etherton TD (2002). Bioactive compounds in foods: their role in the prevention of cardiovascular disease and cancer. Am J Med.

[2] Biesalski HK, Dragsted LO, Elmadfa I, Grossklaus R, Muller M, Schrenk D, Walter P, Weber P (2009). Bioactive compounds: definition and assessment of activity. Nutrition.

[3] Ellwood K, Balentine DA, Dwyer JT, Erdman JW, Gaine PC, Kwik-Uribe CL (2014). Considerations on an approach for establishing a framework for bioactive food components. Adv Nutr.

[4] Putnam KP, Bombick DW, Doolittle DJ (2002). Evaluation of eight *in vitro* assays for assessing the cytotoxicity of cigarette smoke condensate. Toxicol in Vitro.

[5] Rein MJ, Renouf M, Cruz-Hernandez C, Actis-Goretta L, Thakkar SK, da Silva Pinto M (2013). Bioavailability of bioactive food compounds: a challenging journey to bioefficacy. Br J Clin Pharmacol.

[6] Kay CD, Kroon PA, Cassidy A (2009). The bioactivity of dietary anthocyanins is likely to be mediated by their degradation products. Mol Nutr Food Res.

[7] Kim W, McMurray DN, Chapkin RS (2010). n-3 polyunsaturated fatty acids – physiological relevance of dose. Prostaglandins Leukot Essent Fatty Acids.

[8] Goya L, Delgado-Andrade C, Rufián-Henares JA, Bravo L, Morales FJ (2007). Effect of coffee melanoidin on human hepatoma HepG2 cells. Protection against oxidative stress induced by *tert*-butylhydroperoxide. Mol Nutr Food Res.

[9] Ramos S, Alía M, Bravo L, Goya L (2005). Comparative effects of food-derived polyphenols on the viability and apoptosis of a human hepatoma cell line (HepG2). J Agric Food Chem.

[10] Purcell R, Latham SH, Botham KM, Hall WL, Wheeler-Jones CP (2014). High-fat meals rich in EPA plus DHA compared with DHA only have differential effects on postprandial lipemia and plasma 8-isoprostane F2a concentrations relative to a control high-oleic acid meal: a randomized controlled trial. Am J Clin Nutr.

[11] Jumpsen JA, Brown NE, Thomson AB, Paul Man SF, Goh YK, Ma D, Clandinin MT (2006). Fatty acids in blood and intestine following docosahexaenoic acid supplementation in adults with cystic fibrosis. J Cyst Fibros.

[12] Kuriki K, Nagaya T, Tokudome Y, Imaeda N, Fujiwara N, Sato J, Goto C, Ikeda M, Maki S, Tajima K (2003). Plasma concentrations of (n-3) highly unsaturated fatty acids are good biomarkers of relative dietary fatty acid intakes: a cross-sectional study. J Nutr.

[13] Lara JJ, Economou M, Wallace AM, Rumley A, Lowe G, Slater C, Caslake M, Sattar N, Lean ME (2007). Benefits of salmon eating on traditional and novel vascular risk factors in young, non-obese healthy subjects. Atherosclerosis.

[14] Jans JJ, de Sain-van der Velden MG, van Hasselt PM, van den Hurk DT, Vaz FM, Visser G, Verhoeven-Duif NM (2013). Supplementation with a powdered blend of PUFAs normalizes DHA and AA levels in patients with PKU. Mol Genet Metab.

[15] Queenan KM, Stewart ML, Smith KN, Thomas W, Fulcher RG, Slavin JL (2007). Concentrated oat b-glucan, a fermentable fiber, lowers serum cholesterol in hypercholesterolemic adults in a randomized controlled trial. Nutr J.

[16] Garland SH (2011). Short chain fatty acids may elicit an innate immune response from preadipocytes: a potential link between bacterial infection and inflammatory diseases. Med Hypotheses.

[17] Al-Lahham SH, Peppelenbosch MP, Roelofsen H, Vonk RJ, Venema K (1801). Biological effects of propionic acid in humans; metabolism, potential applications and underlying mechanisms. Biochim Biophys Acta.

[18] Al-Lahham SH, Roelofsen H, Priebe M, Weening D, Dijkstra M, Hoek A, Rezaee F, Venema K, Vonk RJ (2010). Regulation of adipokine production in human adipose tissue by propionic acid. Eur J Clin Investig.

[19] Anson NM, Havenaar R, Vaes W, Coulier L, Venema K, Selinheimo E, Bast A, Haenen GR (2011). Effect of bioprocessing of wheat bran in wholemeal wheat breads on the colonic SCFA production *in vitro* and postprandial plasma concentrations in men. Food Chem.

[20] Cummings JH, Pomare EW, Branch WJ, Naylor CP, Macfarlane GT (1987). Short chain fatty acids in human large intestine, portal, hepatic and venous blood. Gut.

[21] Nilsson AC, Östman EM, Knudsen KE, Holst JJ, Björck IM (2010). A cereal-based evening meal rich in indigestible carbohydrates increases plasma butyrate the next morning. J Nutr.

[22] Tarini J, Wolever TM (2010). The fermentable fibre inulin increases postprandial serum short-chain fatty acids and reduces free-fatty acids and ghrelin in healthy subjects. Appl Physiol Nutr Metab.

[23] Meijer K, de Vos P, Priebe MG (2010). Butyrate and other short-chain fatty acids as modulators of immunity: what relevance for health?. Curr Opin Clin Nutr Metab Care.

[24] de Pascual-Teresa S, Sanchez-Ballesta M (2008). Anthocyanins: from plant to health. Phytochem Rev.

[25] de Ferrars RM, Czank C, Zhang Q, Botting NP, Kroon PA, Cassidy A, Kay CD (2014). The pharmacokinetics of anthocyanins and their metabolites in humans. Br J Pharmacol.

[26] Frank T, Janssen M, Netzet G, Christian B, Bitsch I, Netzel M (2007). Absorption and excretion of elderberry (*Sambucus nigra* L.) anthocyanins in healthy humans. Methods Find Exp Clin Pharmacol.

[27] Milbury PE, Vita JA, Blumberg JB (2010). Anthocyanins are bioavailable in humans following an acute dose of cranberry juice. J Nutr.

[28] Vitaglione P, Donnarumma G, Napolitano A, Galvano F, Gallo A, Scalfi L, Fogliano V (2007). Protocatechuic acid is the major human metabolite of cyanidin-glucosides. J Nutr.

[29] Czank C, Cassidy A, Zhang Q, Morrison DJ, Preston T, Kroon PA, Botting NP, Kay CD (2013). Human metabolism and elimination of the anthocyanin, cyanidin-3-glucoside: a ^13^C-tracer study. Am J Clin Nutr.

[30] Azzini E, Vitaglione P, Intorre F, Napolitano A, Durazzo A, Foddai MS, Fumagalli A, Catasta G, Rossi L, Venneria E (2010). Bioavailability of strawberry antioxidants in human subjects. Br J Nutr.

[31] McKay DL, Chen CY, Zampariello CA, Blumberg JB (2015). Flavonoids and phenolic acids from cranberry juice are bioavailable and bioactive in healthy older adults. Food Chem.

[32] Russell WR, Scobbie L, Labat A, Duthie GG (2009). Selective bio-availability of phenolic acids from Scottish strawberries. Mol Nutr Food Res.

[33] Weyermann J, Lochmann D, Zimmer A (2005). A practical note on the use of cytotoxicity assays. Int J Pharm.

[34] Riss TL, Moravec RA (2004). Use of multiple assay endpoints to investigate the effects of incubation time, dose of toxin, and plating density in cell-based cytotoxicity assays. Assay Drug Dev Technol.

[35] Chirakkal H, Leech SH, Brookes KE, Prais AL, Waby JS, Corfe BM (2006). Upregulation of BAK by butyrate in the colon is associated with increased Sp3 binding. Oncogene.

[36] Franken NA, Rodermond HM, Stap J, Haveman J, van Bree C (2006). Clonogenic assay of cells *in vitro*. Nat Protoc.

[37] Griffiths GJ, Corfe BM, Savory P, Leech S, Esposti MD, Hickman JA, Dive C (2001). Cellular damage signals promote sequential changes at the N-terminus and BH-1 domain of the pro-apoptotic protein Bak. Oncogene.

[38] Salakou S, Kardamakis D, Tsamandas AC, Zolota V, Apostolakis E, Tzelepi V, Papathanasopoulos P, Bonikos DS, Papapetropoulos T, Petsas T (2007). Increased Bax/Bcl-2 ratio up-regulates caspase-3 and increases apoptosis in the thymus of patients with myasthenia gravis. In Vivo.

[39] Di Nunzio M, van Deursen D, Verhoeven AJ, Bordoni A (2010). n-3 and n-6 polyunsaturated fatty acids suppress sterol regulatory element binding protein activity and increase flow of non-esterified cholesterol in HepG2 cells. Br J Nutr.

[40] Valli V, Gomez-Caravaca AM, Di Nunzio M, Danesi F, Caboni MF, Bordoni A (2012). Sugar cane and sugar beet molasses, antioxidant-rich alternatives to refined sugar. J Agric Food Chem.

[41] Gartlon J, Kinsner A, Bal-Price A, Coecke S, Clothier RH (2006). Evaluation of a proposed *in vitro* test strategy using neuronal and non-neuronal cell systems for detecting neurotoxicity. Toxicol in Vitro.

[42] O'Brien J, Wilson I, Orton T, Pognan F (2000). Investigation of the Alamar Blue (resazurin) fluorescent dye for the assessment of mammalian cell cytotoxicity. Eur J Biochem.

[43] Danesi F, Ferioli F, Caboni MF, Boschetti E, Di Nunzio M, Verardo V, Valli V, Astolfi A, Pession A, Bordoni A (2011). Phytosterol supplementation reduces metabolic activity and slows cell growth in cultured rat cardiomyocytes. Br J Nutr.

[44] Buch K, Peters T, Nawroth T, Sanger M, Schmidberger H, Langguth P (2012). Determination of cell survival after irradiation via clonogenic assay versus multiple MTT assay--a comparative study. Radiat Oncol.

[45] Rasband WS (1997-2016) ImageJ. U.S. National Institutes of Health. https://imagej.nih.gov/ij/. Accessed 1 June 2016.

[46] Sakurazawa T, Ohkusa T (2005). Cytotoxicity of organic acids produced by anaerobic intestinal bacteria on cultured epithelial cells. J Gastroenterol.

[47] Tang Y, Chen Y, Jiang H, Nie D (2011). Short-chain fatty acids induced autophagy serves as an adaptive strategy for retarding mitochondria-mediated apoptotic cell death. Cell Death Differ.

[48] Chen PN, Chu SC, Chiou HL, Chiang CL, Yang SF, Hsieh YS (2005). Cyanidin 3-glucoside and peonidin 3-glucoside inhibit tumor cell growth and induce apoptosis *in vitro* and suppress tumor growth *in vivo*. Nutr Cancer.

[49] Hidalgo M, Martin-Santamaria S, Recio I, Sanchez-Moreno C, de Pascual-Teresa B, Rimbach G, de Pascual-Teresa S (2012). Potential anti-inflammatory, anti-adhesive, anti/estrogenic, and angiotensin-converting enzyme inhibitory activities of anthocyanins and their gut metabolites. Genes Nutr.

[50] Williamson G, Clifford MN (2010). Colonic metabolites of berry polyphenols: the missing link to biological activity?. Br J Nutr.

[51] Aragonès G, Danesi F, Del Rio D, Mena P. The importance of studying cell metabolism when testing the bioactivity of phenolic compounds. Trends Food Sci Technol. 2017; doi: 10.1016/j.tifs.2017.02.001.

[52] Andrade LN, de Lima TM, Curi R, Castrucci AM (2005). Toxicity of fatty acids on murine and human melanoma cell lines. Toxicol in Vitro.

[53] Ding WQ, Vaught JL, Yamauchi H, Lind SE (2004). Differential sensitivity of cancer cells to docosahexaenoic acid-induced cytotoxicity: the potential importance of down-regulation of superoxide dismutase 1 expression. Mol Cancer Ther.

[54] Kang KS, Wang P, Yamabe N, Fukui M, Jay T, Zhu BT (2010). Docosahexaenoic acid induces apoptosis in MCF-7 cells *in vitro* and *in vivo* via reactive oxygen species formation and caspase 8 activation. PLoS One.

[55] Langelier B, Linard A, Bordat C, Lavialle M, Heberden C (2010). Long chain-polyunsaturated fatty acids modulate membrane phospholipid composition and protein localization in lipid rafts of neural stem cell cultures. J Cell Biochem.

[56] Jing K, Song KS, Shin S, Kim N, Jeong S, Oh HR, Park JH, Seo KS, Heo JY, Han J (2011). Docosahexaenoic acid induces autophagy through p53/AMPK/mTOR signaling and promotes apoptosis in human cancer cells harboring wild-type p53. Autophagy.

[57] Healy DA, Watson RW, Newsholme P (2003). Polyunsaturated and monounsaturated fatty acids increase neutral lipid accumulation, caspase activation and apoptosis in a neutrophil-like, differentiated HL-60 cell line. Clin Sci.

[58] D'Eliseo D, Velotti F (2016). Omega-3 fatty acids and cancer cell cytotoxicity: implications for multi-targeted cancer therapy. J Clin Med..

[59] Di Nunzio M, Valli V, Bordoni A (2011). Pro- and anti-oxidant effects of polyunsaturated fatty acid supplementation in HepG2 cells. Prostaglandins Leukot Essent Fatty Acids.

[60] Righi V, Di Nunzio M, Danesi F, Schenetti L, Mucci A, Boschetti E, Biagi P, Bonora S, Tugnoli V, Bordoni A (2011). EPA or DHA supplementation increases triacylglycerol, but not phospholipid, levels in isolated rat cardiomyocytes. Lipids.

[61] Jaureguiberry MS, Tricerri MA, Sanchez SA, Finarelli GS, Montanaro MA, Prieto ED, Rimoldi OJ (2014). Role of plasma membrane lipid composition on cellular homeostasis: learning from cell line models expressing fatty acid desaturases. Acta Biochim Biophys Sin.

[62] Schumann J, Leichtle A, Thiery J, Fuhrmann H (2011). Fatty acid and peptide profiles in plasma membrane and membrane rafts of PUFA supplemented RAW264.7 macrophages. PLoS One.

[63] Masella R, Santangelo C, D'Archivio M, Li Volti G, Giovannini C, Galvano F (2012). Protocatechuic acid and human disease prevention: biological activities and molecular mechanisms. Curr Med Chem.

[64] Vauzour D, Tejera N, O'Neill C, Booz V, Jude B, Wolf IMA, Rigby N, Silvan JM, Curtis PJ, Cassidy A (2015). Anthocyanins do not influence long-chain n-3 fatty acid status: studies in cells, rodents and humans. J Nutr Biochem.

[65] Ghini V, Di Nunzio M, Tenori L, Valli V, Danesi F, Capozzi F, Luchinat C, Bordoni A (2017). Evidence of a DHA signature in the lipidome and metabolome of human hepatocytes. Int J Mol Sci.

[66] Schwenk RW, Holloway GP, Luiken JJ, Bonen A, Glatz JF (2010). Fatty acid transport across the cell membrane: regulation by fatty acid transporters. Prostaglandins Leukot Essent Fatty Acids.

[67] Moschen I, Bröer A, Galić S, Lang F, Bröer S (2012). Significance of short chain fatty acid transport by members of the monocarboxylate transporter family (MCT). Neurochem Res.

[68] Björk C, Wilhelm U, Mandrup S, Larsen BD, Bordoni A, Hedén P, Rydén M, Arner P, Laurencikiene J (2016). Effects of selected bioactive food compounds on human white adipocyte function. Nutr Metab.

[69] Fotakis G, Timbrell JA (2006). *In vitro* cytotoxicity assays: comparison of LDH, neutral red, MTT and protein assay in hepatoma cell lines following exposure to cadmium chloride. Toxicol Lett.

